# Exploring Attachment and Internal Representations in Looked-After Children

**DOI:** 10.3389/fpsyg.2020.00464

**Published:** 2020-03-19

**Authors:** Saul Hillman, Richard Cross, Katharine Anderson

**Affiliations:** ^1^The Anna Freud National Centre for Children and Families, Kantor Centre of Excellence, London, United Kingdom; ^2^Assessment and Therapy, Five Rivers Child Care Limited, Salisbury, United Kingdom

**Keywords:** attachment, story stems, narrative tasks, looked-after children, fostering, residential, maltreatment, internal representations

## Abstract

**Background:**

This article explores the Story Stem Assessment Profile (SSAP), a narrative-based measure, for the assessment of internal representations in children between the ages of 4 and 11 years old.

**Methods:**

The findings draw upon two samples of children comprising of a sample of looked-after children at Five Rivers Child Care (FR) (*n* = 42) and a community-based population (*n* = 42). The FR group identified were suggested to have a higher level of need, as defined by scores obtained from the Strengths and Difficulties Questionnaire (SDQ) and Relationship Problems Questionnaire (RPQ).

**Results:**

Using the SSAP, the findings indicate the instrument’s discriminant validity with strong differences being displayed between the two populations. Consistently children in the FR sample displayed more disorganized, avoidant and negative representations, whilst at the same time having significantly fewer representations characteristic of ‘secure’ attachment.

**Conclusion:**

The SSAP is successful in differentiating between ‘low’ and ‘high’ cohorts of children aged 4–11 years. The study provides strong support for the measure as a way of capturing internal and attachment representations, with further research to explore possible changes in these representations at follow-up being promising and intriguing. Continued research efforts at FR will allow for improved clinical formulations, increased understanding and therefore positive outcomes relating to the children in their care.

## Introduction

Foster and residential care placements provide children who have had prior experiences of abuse and/or neglect with an alternative, protective care environment (McGrath-Lone et al., 2016). Figures approximate there are currently 78,150 children who are looked-after in England, with one in thirty children having experienced foster care by the time they are 18 years old (Department for Education, 2019). Children who become looked-after are under the care of the local authority; children may enter the care system voluntarily, or if removed from their birth family by a court order (McGrath-Lone et al., 2016). Generally, children will be placed in residential care if other placement options, such as foster care, have been unsuccessful or unable to meet the child’s level of need (Strijbosch et al., 2015).

Looked-after children come from varying backgrounds, having experienced a diverse range of often adverse early life experiences (Morgan and Baron, 2011), with 92% of children being placed in care due to abuse, neglect, family dysfunction or absent parenting (Department for Education, 2019). Unfortunately, these experiences are often relationally traumatic, which impacts upon a child’s ability to form positive attachments to their caregiver(s).

Attachment is defined as a deep and enduring emotional bond which connects one person to another (Ainsworth, 1973). A child will seek attachment to their caregiver(s), for emotional regulation, attunement, to develop emotional intelligence and, evolutionary speaking, for survival (Schore, 2001a). Children are vulnerable and highly dependent; therefore, they invest in connecting to an adult who can guide and support their development (Howe, 2005). Children who end up in care need to be met with a relationship which provides the love, safety and nurture they need. Otherwise, the child is likely to be faced with a caregiver and environment which leaves them feeling unloved, rejected, angry or confused (Bowlby, 1969).

Henry (1974) describes how children hold beliefs of their past relationships and repeat them with other caring adults. Furthermore, children who have suffered adversity are likely to have experienced care-giving environments which are avoidant or ambivalent, with potential exposure to abandonment and unpredictable danger (Howe, 2006). At a young age, a child develops an internal working model (IWM): a cognitive framework which influences how the child understands the world, the self and others (Bowlby, 1969). A child’s attachment relationship with their caregiver will heavily influence how their IWM develops (Bowlby, 1969). Children in care are likely to have established IWM’s based on their early experiences of adversity, which they have learnt and understood as a replica for future relationships (Bowlby, 1969).

In relation to attachment security, children in care have been shown to have more insecure and disorganized attachment styles (Zaccagnino et al., 2015). If a child experiences their caregiver as unwilling (avoidant) or unable (ambivalent), the child will have to reorganize and adapt their attachment needs; the suppressing of which distorts the child’s ability to make sense of their emotions, thoughts and behaviors (Howe, 2005). This is intensified for children who also experience frightening, abusive or hostile behavior at the hands of their caregiver. A disorganized attachment will likely develop, in which the child starts to adapt and synchronize with their caregivers dysregulated and distressing behavior (Lyons-Ruth, 1996; Schore, 2001b).

Additional research from Cyr et al. (2010) who examined the impact of experiences of maltreatment on attachment security and organization, found children living in high risk environments were more likely to develop insecure and disorganized attachment styles. Similarly, Stronach et al. (2011) found early childhood maltreatment to be associated with more negative representations of the mother–child relationship. Therefore, the child is often left striving for survival, desperately trying to establish a sense of order and safety within their relationships (Howe, 2006).

When referring to disorganized attachment, theorists have suggested for infants there exists an “irresolvable paradox” in their IWM, in that their initial caregiver, who should be a source of safety and protection, is simultaneously being identified as a source of danger or harm (Cicchetti, 1990; Hildyard and Wolfe, 2002). Children who experience this kind of caregiving are likely to respond in the same way to a new caregiver, as though they are the original, potentially threatening adult from their birth family (Howe, 2006). If this is not recognized and understood by the new adults caring for the child in their foster or residential care placement, this can result in “double deprivation” (Henry, 1974), whereby a child experiences difficulty within relationships, often culminating in repeated rejection through frequent placement moves and disrupted relationships with their new caregivers. Behaviors which were adaptive within an abusive environment may cause complications in the formation of new relationships (Main and George, 1985; Waldinger et al., 2001).

Due to the above lasting implications of attachment on relationships, a vast amount of research has explored various methods to measure attachment and associated factors for looked-after children. The Narrative Story Stem Technique (NSST; Page, 1998) was developed to create a window of insight into a child’s internal world and IWM. Within the NSST, systematic sets of story beginnings are acted out in play with a child (Emde et al., 2003). A child will be introduced to a story (the stem) containing an unresolved tension, with their task being to finish the story and address the dilemma. Research has shown the effectiveness of using the NSST as a semi-projective measure to elicit themes about conflicts, internal representations of relationships and family dynamics (Buchsbaum et al., 1992). From the age of three, narrative-based techniques have been found successful in exploring attachment, with children being able to provide resolutions to attachment-based stories (Bretherton et al., 1990).

Using the story stem technique, research has shown that children who experience abusive or insensitive caregiving are more likely to display insecure attachment representations within their stories (Huetteman, 2005). This is represented in their dialogue and actions by portraying negative representations of their self, and of their caregivers (Cicchetti et al., 1995; Grych et al., 2002; Minnis et al., 2006b). Studies have also found that maltreated children display more avoidant behavior in their narratives and may minimize their responses (Toth et al., 2000). There may also be a failure in acknowledging conflicts presented in the story stem, as well as a lack of resolution or reparation of the dilemma (Buchsbaum et al., 1992).

Venet et al. (2007) explored the internal attachment representations of a group of thirty-nine pre-school children who had experienced neglect, in comparison with a control sample who had not. Significant differences in representations were found between the two groups, with significantly higher levels of avoidant attachment present in the group of children who had experienced neglect. This group also displayed greater traits of disorganization, with negative depictions of their mothers being represented in their stories.

The Story Stem Assessment Profile (SSAP) developed at the Anna Freud National Centre for Children and Families (AFNCCF) has been widely used to examine attachment and internal representations of looked-after children (Hodges et al., 2000). Children in care have been found to exhibit higher levels of disorganization, defensive-avoidance, and insecure constructs, demonstrating less qualities associated with secure attachment (Hillman et al., 2016). When the SSAP was used with a sample of internationally adopted children in contrast to a control sample of children living at home with their birth parents, the findings showed the adopted group displayed more negative representations (Román et al., 2013). It was suggested that negative representations were likely to be related to the long-term impact of early adverse childhood experiences suffered in the adopted group (Román et al., 2013). The findings using the SSAP reinforce outcomes from previous research, demonstrating how maltreated children’s stories were less coherent, with more negative and conflictual comment (Toth et al., 1997; Minnis et al., 2006a).

The present study wishes to further explore the use of the SSAP in identifying attachment and internal representations of looked-after children. The sample drawn consists of children in both fostering and residential care at Five Rivers Child Care (FR), matched with a community based (COMM) sample of children living at home with their birth parents. In comparison to the FR sample, the COMM sample had not experienced any discontinuities in care, no known neglect or maltreatment, and no known contact with clinical or social services for any mental health difficulties. The study considers and draws upon several background variables of the looked-after cohort, obtained through carer report. It is hoped the output of the present study will help to support care staff and carers at Five Rivers to further understand attachment and internal representations of children who have been exposed to abuse and/or neglect and provide a framework for intervention. The study also examines the differences between FR and COMM cohorts on carers reports of children’s behavior. This allows for a more external measure of a child’s psychopathology in contrast to a child-led measure of internal and attachment representations.

The study draws upon three hypotheses:

(1) Children in the FR sample will have lower scores on the ‘secure’ SSAP construct and higher scores on ‘defensive avoidant,’ ‘insecure,’ and ‘disorganized.’
(2) Children in the COMM sample will have the highest scores on the ‘secure’ SSAP construct and the lowest scores on ‘defensive avoidant,’ ‘insecure’ and ‘disorganized.’
(3) Children in the FR sample will display higher scores on the carers report across Total Behavior and associated subscales than those in the COMM sample (see measures).

## Materials and Methods

The present study was a collaborative effort between Five Rivers Child Care (FRCC), a national fostering, residential, educational and assessment and therapy service based in Salisbury and the Anna Freud Centre for Children and Families (AFNCCF), London. The purpose was to explore what the internal world of looked-after children may look like by using the Story Stem Assessment Profile (SSAP) (Hodges et al., 2000). The specific locus of interest was how the cohort view the self and their representations of others. The study compares data from the Five Rivers (FR) sample of looked after children with a matched sample of non-maltreated children (COMM) who live with their biological parents.

All carers at FR complete a set of standard self-report measures aiming to explore the emotional and behavioral wellbeing of the children in their care. Recent developments have enabled the SSAP to be administered to children aged 4–11 years old at Five Rivers who have scores of clinical concern across either/or the Strengths and Difficulties Questionnaire (SDQ; Goodman, 1997) and Relationship Problems Questionnaire (RPQ; Minnis et al., 2002, 2007). For the SDQ, a score of 17 or above indicates high cause for concern, with 6 or 7 being the marker of clinical need within the RPQ.

### Participants

A total sample of 84 was gathered; matched evenly on gender and age. Males and females were equally distributed in each sample (50%); see Table 1. Of the 42 children drawn from FR, 5 children (11.4%) were in residential care, and 39 (88.6%) were in foster care. The COMM sample (*n* = 42) was formed through mainstream primary schools in London and the South-East and were considered a ‘low-risk’ sample, with children living within their birth families. There were no known previous maltreatment or discontinuities and no direct contact with clinical services in relation to the child. In the FR sample, pre-placement discontinuities varied with 32.4% having no previous placements, 48.6% having between 1 and 2 placements and 18.9% having three or more. Previous maltreatment was noted in all children in the FR cohort (100% emotional abuse, 45% neglect, 31% domestic violence, 26% physical abuse, 24% sexual abuse, with concerns over parental capacity [e.g., mental health difficulties, alcohol/substance misuse] being report in 65% of cases).

**TABLE 1:** Gender matching of participants in both the FR and COMM cohorts.

	FR (*n* = 40)	COMM (*n* = 45)
Age	*M* = 85.32, *SD* = 18.41	*M* = 83.97, *SD* = 13.89
Gender	*M* = 19, Female = 19	*M* = 19, Female = 19

### Descriptive Statistics

Children in the FR sample ranged in age from 44 to 121 months (*M* = 84.58 months, *SD* = 19.16), with children in the COMM sample varying from 48 to 106 months (*M* = 83.98 months, *SD* = 13.89. The age of the two groups did not significantly differ (*t* = 1.10, 88, *p* = 0.158).

### Measures

The Story Stem Assessment Profile (SSAP; Hodges et al., 2000, 2007) entails a set of 13 story stems, which introduce the beginning of a story for the child to complete, within which lies “an inherent dilemma.” It “allows an assessment of the child’s expectations and perceptions of family roles without asking the child direct questions about his or her own family which might cause him or her undue conflict or anxiety” (Goodman and Scott, 1999). Five of the stories were developed by Hodges (1992) based on her clinical experience in assessing abused children, with the remaining eight stems selected from the MacArthur Story Stem Battery (MSSB; Bretherton et al., 1990), which had been previously employed in non-clinical situations. A shortened version of the SSAP consisting of seven stories was adopted for this study, which had demonstrated robust psychometric properties (SSAP7; Hillman, 2011).

Each story stem from a completed set is coded on 39 individual codes (themes) by assigning a score of 0 (the absence of a theme), 1 (the ambiguity or lesser appearance of a theme, or following a prompt), or 2 (the definite or stronger appearance of a theme). The themes are organized into four constructs: security, insecurity, defensive avoidance and disorganization.

Strength and Difficulties Questionnaire (SDQ; Goodman, 1997) was developed in the United Kingdom to measure emotional and behavioral well-being. The questionnaire is administered to parents and teachers of the children and comprises of 25 items, each with a five-point scale. The items come under five different attributes consisting of *emotional symptoms, conduct difficulties, hyperactivity/inattention, peer relationship problems*, and *pro-social behavior*. Research by Goodman and Scott (1999) has evidenced predictive validity for the use of the SDQ for children from both clinical and normative populations.

The Relationship Problem Questionnaire (RPQ; Minnis et al., 2007) is a 10-item screening instrument for reactive attachment disorder (RAD). This measure includes two subscales: disinhibited and inhibited behavior. Carers rate the child on a 4-point Likert scale from 0 (not like my child) to 3 (exactly like my child). The possible score range in 0–20. 6 and 7 are two recommended cut off scores for a likely diagnosis of RAD (Minnis et al., 2013).

### Procedure

The project was initially conceived to shed further light on the story stem data that had been collected at Five Rivers. In recruiting participants for this study, the Five Rivers Assessment and Therapy (A&T) team identified a possible sample of children in foster and residential care with high, clinical scores on either or both the SDQ and the RPQ. In the FR sample, 81.3% had scores above the clinical cut off on the SDQ, while 18.7% had scores below this. On the RPQ, 57.6% were about the clinical cut off, while 42.4% fell below. Those who did not score above the cut off points were included due to other determining factors, e.g., presentation in placement, further need to understand the child’s attachment style.

A letter of informed consent was sent to each child’s local authority social worker and their FR supervising social worker, requesting should they wish for the child not to participate in the assessment to notify the A&T team within 2 weeks. The opt-out date was clearly stated in the letter. Due to the age of the children, informed consent was provided by their delegated authority figures(s), however, it was made clear that should a child become distressed or wish to withdraw from the assessment, they were ethically within their right to do so.

Once consent was obtained, a detailed information sheet about the story stem assessment and a frequently asked questions document was sent to the child carers. The carers were required to give their informed consent also.

Following this, the trained clinicians at Five Rivers administered the shortened SSAP (SSAP7; Hillman, 2011) in the family or residential home. The SDQ was completed by the foster carers and retuned alongside additional measures which are not reported within this study. The SSAP assessments were audio-recorded upon administration, and audio files for the broader Five Rivers group were transcribed by postgraduate researchers/students, with support from the A&T team. The data was coded by the author with 20 per cent coded by two additional postgraduate researchers. Coding of the SSAP was carried out by postgraduate students, who were blind to the samples from which the reliability cases came, and validation of these were ensured by an accredited trainer of the SSAP and primary author of this paper (*k* = 0.82, 0.69 < *k* < 1.00).

### Ethics Statement

Ethical approval was obtained through University College London. Children’s story stems were audio-recorded and adhered to the Data Protection Act (Department for Digital, Culture, Media and Sport, 2018), ensuring all data remained anonymous, stored securely and individuals were not able to be identified. Though the SSAP was a semi-projective task that did not involve any direct questions, precautions were adopted by the researcher in case any specific storm stem elicited a negative response in the participant, in which case the story and recording was stopped.

### Planned Analysis

A range of descriptive and inferential statistics (SPSS: version 25.0) were used to examine for differences in the SSAP and SDQ between both the FR and COMM sample.

## Results

### SSAP Constructs

Initially, four independent t-tests were conducted on the four SSAP constructs (Secure, Insecure, Disorganized, Defensive-Avoidant) between the FR and COMM samples.

Table 2 shows how the four constructs differentiated the two samples. In each case, there was a statistically significant difference. The ‘Secure’ construct was significantly higher in the COMM sample [*t*(88) = 3.08, *p* < *0.01*; *d* = 0.65] with ‘Insecure’ scores being statistically higher in the FR sample [*t*(88), −6.81, *p* < 0.001; *d* = 1.45]. ‘Disorganized’ scores were statistically higher in the FR sample [*t*(88), −7.71, *p* < 0.001; *d* = 1.91] and finally, ‘Defensive-Avoidant’ scores were significantly higher in the FR sample [*t*(88), −6.55, *p* < 0.001; *d* = 1.87]. The effect size for three of these constructs were very large and exceeded Cohen’s (1988) convention for a large effect (*d* = 0.80).

**TABLE 2:** SSAP Construct Differences between the two samples.

	FR	COMM
Secure **	3.13 (1.91)	4.36 (1.87)
Insecure ***	2.89 (1.83)	0.98 (0.54)
Disorganized ***	2.67 (1.57)	0.68 (0.74)
Def-Avoidant ***	3.17 (2.63)	0.58 (0.55)

Means are in each cell (SDs in parentheses). ** *p* < 0.01; *** *p* < 0.001.

The four SSAP constructs all significantly differentiated between the two populations. In all cases, the FR sample were significantly lower on *secure* representations whilst much higher on *defensive-avoidant*, insecure and *disorganized* representations.

It was also felt important to explore these representations in more detail to see whether the individual SSAP codes or themes were also differentiating the samples. First, the set of ‘child representations’ were examined (see Table 3).

**TABLE 3:** Differences between FR and COMM: Child Representation codes.

	FR	COMM	T statistic Equality Means			
Child Representations			*t*	df	*p*	*d*
Child Seeks Help ***	0.10 (0.18)	0.47 (0.24)	7.21	88	<0.001	1.68
Siblings/Peers Help **	0.17 (0.22)	0.47 (0.24)	6.08	88	<0.01	1.29
Realistic Mastery **	0.20 (0.19)	0.33 (0.22)	3.02	88	<0.01	0.64
Acknowledge Distress	0.40 (0.34)	0.36 (0.32)	−0.61	88	0.242	0.13
Child Endangered **	0.63 (0.55)	0.26 (0.25)	−4.19	88	<0.01	1.03
Child Injured/Dead *	0.53 (0.54)	0.31 (0.21)	−2.55	88	<0.05	0.65
Excessive Compliance **	0.19 (0.24)	0.05 (0.10)	−3.83	88	<0.01	1.00
Child Parents/Controls *	0.18 (0.30)	0.08 (0.10)	−2.18	88	<0.05	0.58
Child Aggresses *	0.46 (0.50)	0.23 (0.24)	−2.80	88	<0.01	0.69

Means are in each cell (SDs in parentheses) * *p* < 0.05; ** *p* < 0.01; *** *p* < 0.001.

Table 3 indicates the FR sample are showing significantly more representations of ‘*child endangered*,’ ‘*child injured/dead*,’ ‘*excessive compliance*,’ ‘*child aggresses*’ and ‘*child parents/controls*,’ whilst showing significantly less positive representations of ‘*child seeks help*,’ ‘*siblings/peers help*,’ and ‘*realistic active mastery.*’ The effect size was large for *‘child seeks help,’ ‘siblings/peers help,’* and *‘excessive compliance,’* while the remaining differences had a moderate effect. There is a clear differentiation between the two samples in terms of how the child is being represented, with far more negative and far fewer positive representations in the FR sample.

Next, Table 4 indicates how ‘adult representations’ are being differentiated between the two populations. The FR sample are showing significantly more representations of ‘*adults unaware*,’ ‘*adults actively rejecting*,’ ‘*adults injured/dead*,’ and ‘*adult aggresses.*’ Furthermore, there were less representations of ‘*adult helps’* and ‘*adult affectionate’* compared to the COMM sample. Three of the codes showed a strong effect size (exceeding 0.80) – *‘adult actively reject,’* ‘*adult injured/dead,’* and ‘*adult aggresses.’* The remaining codes remained non-significant. As with the child representation, there is a clear distinction between the two samples in terms of how the adult (carer) figures are being represented with far more negative and far fewer positive representations in the FR sample.

**TABLE 4:** Differences between FR and COMM: Adult Representation codes.

	FR	COMM	T statistic Equality Means			
Adult Representations			*t*	df	*p*	*d*
Adult Comforts	0.14 (0.22)	0.21 (0.22)	1.42	88	0.145	0.30
Adult Helps *	0.57 (0.36)	0.76 (0.35)	2.50	88	<0.05	0.53
Adult Affectionate *	0.28 (0.30)	0.39 (0.27)	1.86	88	<0.05	0.39
Acknowledge Distress	0.15 (0.29)	0.20 (0.18)	0.90	88	0.459	0.19
Limit Setting	0.65 (0.45)	0.56 (0.33)	−1.02	88	0.115	0.22
Adult Unaware ***	0.41 (0.22)	0.15 (0.13)	−6.85	88	<0.001	1.60
Adult Rejects **	0.22 (0.30)	0.05 (0.13)	−3.54	88	<0.01	0.90
Adult Injured/Dead **	0.41 (0.46)	0.11 (0.15)	4.21	88	<0.01	1.14
Adult Aggresses **	0.66 (0.60)	0.28 (0.26)	3.94	88	<0.01	1.00

Means are in each cell (SDs in parentheses). * *p* < 0.05; ** *p* < 0.01; *** *p* < 0.001.

The more ‘disorganized’ clusters of codes were examined in relation to the two populations (see Table 5). Here, there is a strong and consistent picture with the FR sample showing more indicators of all the themes – higher levels of ‘*extreme aggression*,’ ‘*catastrophic fantasy*,’ ‘*bizarre atypical material*,’ ‘*bad to good shifts*,’ ‘*magic omnipotence*,’ and ‘*no closure.*’ All these codes had large effect sizes.

**TABLE 5:** Differences between FR and COMM: disorganization codes.

	FR	COMM	T statistic Equality Means			
Disorganization Reps			*t*	df	*p*	*d*
Extreme Aggression ***	0.56 (0,64)	0.12 (0.18)	−4.47	88	<0.001	1.23
Catastrophic Fantasy **	0.52 (0.60)	0.10 (0.18)	−4.56	88	<0.001	1.25
Bizarre Material ***	0.78 (0.63)	0.18 (0.23)	−6.05	88	<0.01	1.60
Bad < > Good Shift ***	0.17 (0.21)	0.02 (0.05)	−4.90	88	<0.001	1.39
Magic Omnipotence **	0.46 (0.51)	0.12 (0.16)	−4.29	88	<0.01	1.16
No Closure **	0.40 (0.51)	0.06 (0.14)	−4.36	88	<0.01	1.19

Means are in each cell (SDs in parentheses). ** *p* < 0.01; *** *p* < 0.001.

Finally, the more specific avoidance manoeuvres adopted in the stories were examined (see Table 6). Table 6 indicates that the FR sample show far more of all seven of the different type of avoidant representations listed. The effect sizes for all the differences were large.

**TABLE 6:** Differences between FR and COMM: defensive avoidance codes.

	FR	COMM	T statistic Equality Means			
Avoidance Representations			*t*	df	*p*	*d*
Disengagement ***	0.21 (0.26)	0.03 (0.07)	−4.55	88	<0.001	1.26
Initial Aversion *	0.15 (0.32)	0.03 (0.11)	−2.31	88	<0.05	0.61
Premature Foreclosure **	0.59 (0.47)	0.10 (0.25)	−6.19	88	<0.001	1.47
Changes Constraints ***	0.21 (0.21)	0.04 (0.05)	−5.35	88	<0.001	1.38
Avoid Narrative ***	0.62 (0.30)	0.27 (0.26)	−5.88	88	<0.001	1.25
Denies Distress **	0.15 (0.20)	0.02 (0.05)	−4.32	88	<0.01	1.19
Neutralization ***	0.43 (0.34)	0.09 (0.14)	−6.21	88	<0.001	1.57

Means are in each cell (SDs in parentheses). * *p* < 0.05; ** *p* < 0.01; *** *p* < 0.001.

### SDQ Constructs

This paper also set out to explore whether the two populations (FR and COMM) were differentiated by the SDQ, a carer self-report measure which focused on internalizing and externalizing behavior. A reduced number of carers completed this measure, resulting in a lower ‘n’: FR (*n* = 41), COMM (*n* = 31).

In the FR sample, 27 (67.5%) had scores above the clinical cut-off score (17) whilst 13 (33.5%) had scores below this. This contrasted starkly with the COMM sample where only four children (12.9%) had scores above the cut-off and the remaining 27 (87.1%) had scores below the cut-off. There was a significant association between sample and Total SDQ category [χ^2^(1,74) = 21.16, *p* < 0.001].

Table 7 shows that the FR sample have significantly higher scores on the four negative subscales (*Emotional Symptoms, Hyperactivity, Peer problems*, *Conduct Disorder*), whilst they had significantly lower scores on *Prosocial Behavior*. In all cases, the effect size was considered large.

**TABLE 7:** Differences between FR and COMM: SDQ.

	FR	COMM	T statistic Equality Means			
SDQ Subscales			*t*	df	*p*	*d*
SDQ Total ***	19.83 (5.74)	9.26 (6.41)	−8.84	74	<0.001	2.06
SDQ Emotional **	4.29 (2.47)	1.48 (1.86)	−4.89	74	<0.01	1.05
SDQ Conduct ***	5.21 (2.21)	4.77 (1.45)	−6.90	74	<0.001	1.80
SDQ Hyperactivity **	6.59 (2.82)	4.26 (2.92)	−4.32	74	<0.01	0.98
SDQ Peer Problems **	3.88 (2.67)	1.74 (1.91)	−5.22	74	<0.01	1.21
SDQ Prosocial ***	5.83 (2.81)	8.84 (1.10)	7.09	74	<0.001	1.79

Means are in each cell (SDs in parentheses). ** *p* < 0.01; *** *p* < 0.001.

*SDQ Total* was correlated with the four SSAP constructs in order to see whether there were any associations between the two measures. Within the COMM sample, Pearson’s *r* correlations showed no relationships between *Total SDQ* and *SSAP Secure* [*r*(31) = 0.231, *p* = 0.244], *SSAP Insecure* [*r*(31) = −0.096, *p* = 0.466], *SSAP Defensive Avoidant* [*r*(31) = −0.131, *p* = 0.157] and *SSAP Disorganized* [*r*(31) = 0.084, *p* = 0.553]. Within the FR sample, there were no relationships between *Total SDQ* and *SSAP Secure* [*r*(38) = 0.094, *p* = 0.367], *SSAP Insecure* [*r*(38) = −0.074, *p* = 0.315], *SSAP Defensive Avoidant* [*r*(38) = −0.093, *p* = *0.311*] and SSAP Disorganized [*r*(38) = −0.068, *p* = 0487].

In sum, the study has provided strong indication of differences in a sample of looked-after children (FR), who have experienced considerable adversity, compared to a community population (COMM). All four SSAP constructs differentiated the samples in the expected direction. Eight out of the nine codes in the ‘Child Representation’ cluster also differentiated the samples. Six out of the nine codes in the ‘Adult Representation’ cluster also differentiated the samples. The six codes in the ‘Disorganized’ cluster were also far more prevalent in the FR sample. All seven codes in the ‘Defensive-Avoidant’ cluster were also more prevalent in the FR sample. The carer report measure (SDQ) also differentiated the samples on the total score and all subscales. There was no relationship between the child’s internal representations (as measured through the SSAP) and the carer report (SDQ).

## Discussion

### Summary of Findings

The findings of the present study indicated significant differences between the FR and COMM cohorts, in-line with the proposed hypotheses. The FR sample consistently demonstrated characteristics of insecure attachment. On all constructs of the SSAP, the FR sample showed significantly higher scores on the constructs of defensive avoidance, disorganized and insecure, while displaying lower scores on the secure construct. These outcomes were consistent with previous research which indicated looked-after children are more likely to have developed insecure attachment patterns and displays of ambivalence or disorganization (Hopkins, 1990; Cicchetti et al., 1995).

All six codes within the ‘disorganized’ construct were significant, with the FR sample displaying higher traits of measurable disorganization, including catastrophic fantasy, bizarre atypical material, extreme aggression and bad to good shifts. These traits are considered to arise as a result of a lack of coherent organized behavioral strategy, which has not been developed due to the experience of caregivers who are frightening or frightened themselves (Main and Solomon, 1990). A child who aligns with disorganized attachment may experience a distorted ego state, whereby their own internal state is not validated, resulting in low self-esteem and a false sense of self (Steele and Steele, 1994).

There were far more positive and secure representations within the COMM sample compared to the FR sample. When exploring the individual codes and themes of the SSAP, the codes pertained to ‘child representations’ showed significant differences across the samples. Children in the FR sample were more likely to show children endangered, injured, aggressing or excessively compliant within their stories. For the codes clustered around ‘adult representations,’ although there were fewer significant differences, the FR sample still showed high levels of adults being unaware, rejecting and aggressing. These findings support previous research which found negative and abusive interactions with caregivers predisposed children to developing more insecure attachments (Sroufe and Fleeson, 1986).

Finally, all eight codes within the defensive avoidance construct were significant. The output of the SSAP demonstrated avoidance and defensive mechanisms to be higher among the FR sample. Again, this is consistent with prior findings. Early experiences of abuse often encourage a child to avoid contact with attachment figures: a defensive strategy with the function of protecting the self from the possibility of further abuse (Kaufman and Cicchetti, 1989).

On the carer measure (SDQ), the differences were also very strong between the FR and COMM samples with the former showing significantly higher scores on SDQ Total and all the difficulty subscales (hyperactivity, emotional symptoms, peer problems, conduct disorder) and lower scores on the prosocial subscale. The existence of psychopathology in clinical populations has been a well-researched topic. Abuse and neglect are common factors that increase the likelihood of significant behavior difficulties in children. Consequently, it was expected that the total behavioral difficulties were significantly higher in the Five Rivers sample than in the community sample. Findings demonstrated the presence of peer relationship problems as significantly higher. Furthermore, prosocial behaviors came up significantly lower in comparison to the community sample for the initial assessment. In middle childhood, friends become more important and children typically have clear favorites for their peers rather than their carers (Kerns et al., 2006). Therefore, it is unsurprising that peer relationship problems are higher in the FR sample since it is an area of behavior that children are likely to display more prominently, which may explain the lack of prosocial behavior as well. Moreover, anti-social behavior is one of the highest documented outcomes of child abuse (Aber et al., 1985).

To summarize, the findings in this study have mirrored research in the field of maltreated children’s internal representations and attachment. When engaging in a semi-projective measure (the SSAP in this case), children who have experienced troubled parent–child relationships are more likely to tell stories which portray their reflections of their early adversities and relationship dynamics. If considered more avoidant, children may not address the interpersonal issues in narratives by digressing and focusing on irrelevant details or avoiding important story themes. If more aligned with disorganized characteristics, children may constrict their stories by producing incoherent, contradictory, disorganized or chaotic stories (McCrone et al., 1994; Shields et al., 2001).

### Strengths and Implications

The importance of recognizing the attachment needs of children who are looked-after is highly prevalent in research and practice. Children in care are highly vulnerable to emotional and mental health difficulties, often as a result of their early adverse childhood experiences (Millward et al., 2006). Biological evidence demonstrates a child’s brain develops depending on the stimulus provided by their physical social and emotional environment (Perry and Hambrick, 2008). For children who have grown up in environments which are neglectful, frightening or inconsistent, they may not have received the nourishment, care and nurture they need to prosper, develop and grow. This can impact upon neurobiological development, with Teicher et al. (2003) demonstrating there are identifiable differences between the brains of children and adults exposed to early abuse and/or neglect, and those brought up in a positive environment.

Positively, the internal representations a child holds are open to revision throughout childhood, adolescence and adulthood (McConnell and Moss, 2011). Contributing factors that can help a child move from a place of insecurity to security include relationship satisfaction, fewer experiences of negative life events and greater emotional openness (Egeland and Farber, 1984). When a child enters the care system, they need to be met with authentic warmth (Cameron and Maginn, 2008) wherein their carers attune and can be with the child, supporting their needs even if faced with challenging or rejecting behavior. Providing unconditional support, sensitivity, and non-punitive childcaring methods (McConnell and Moss, 2011), the child can be supported to develop a more positive sense of the world, the self and others.

Utilizing the output of the SSAP alongside that of other measures of emotional and behavioral wellbeing can therefore support foster carers and residential staff at Five Rivers to further understand the attachment needs of the children and young people within their care. Assessment information gathered can inform care, help with the development of behavioral support plans and can guide staff to commit to training in attachment to help them acknowledge when their own attachment system is activated, and perhaps when they are experiencing reduced commitment to a child due to experiencing a level of stress (Farmer et al., 2005). All these factors may have a positive impact on placement stability. Having an assessment process that goes beyond that of using only the SDQ (Department for Education and Skills, 2007) allows, contrary to government policy, for a more robust understanding of children in care, who are often largely misunderstood. Furthermore, it ensures that the SDQ is not used alone as a sole means of identifying significant needs in a vulnerable, high-risk population (Wright et al., 2019).

### Limitations

Though the COMM and FR cohorts had similar parameters for gender and age, it was not possible to control for these and other variables more stringently. While the COMM sample was used as a comparison, and children were expected not to have experienced adversity in the past, there was the possibility of unknown factors in both the children’s current and previous experiences. In reference to the FR sample, the number of children from residential care was significantly smaller than those in foster care. This however is a reflection of the number of children in each service (in foster care at FR: 520, in residential care: 13).

A further note of caution must be attached both to the large numbers of tests conducted and to the actual statistical significance that emerged from the analysis. The effect sizes by and large are small to medium despite statistical significance being demonstrated. This suggests the effects on this population are moderate at best. Further studies exploring differences between looked-after and community populations, where children have not experienced adversity and discontinuity, should be encouraged.

An increased sample size would allow far more differentiation of the current population which would enable exploration of some of the more subtle correlates and covariates within these children including age of placement, discontinuities in care and maltreatment. It would also allow us to explore how the child’s internal world, as measured through the SSAP, may relate to carer and practitioners’ measures such as the SDQ, RPQ and Health of the Nations Outcome Scales for Children and Adolescents (HoNOSCA). To date we are not able to understand these relationships given these limitations. There is further potential to qualitatively explore the children’s stories to extract additional material about their perceptions and representations.

Although the SSAP is a measure that accesses both verbal and non-verbal interactions, there is a limitation here. The coding system does not favor verbal narrative responses from non-verbal or enacted responses, however, in the current study, the SSAP was only recorded via audio, not via video recording. The decision was made due to the wish to limit the intrusive nature of the assessment on children who may have experienced significant trauma and abuse. In future research it could be considered whether the collection of cognitive data from participants would be of benefit to help reduce potential confounders in cognitive capacity and verbal ability.

### Future Direction

There is longitudinal potential for this study whereby the SSAP and other measures are currently being repeated in placement at FR at 12 monthly intervals. This promises to answer questions about how children’s internal working models may change over time, and to further examine possible mediators and moderators in the form of interventions or practice which may be explaining this. It is hoped that future research studies on this piece will answer these questions, which would have a vital role in clinical practice and policy in supporting children who are looked-after.

## Conclusion

This paper has presented a very encouraging window into how the FR sample of maltreated and neglected children may present fractured internal working models in comparison to a COMM sample. Strong evidence has been provided that the SSAP, as an instrument of internal working models, manages to differentiate the two samples, showing children in the FR sample to have the least security and the most defensive avoidance, disorganization and insecurity representations.

The results of this study support the notion that systems caring for maltreated children would benefit from a standardized approach to assessing attachment and adversity (Cross, 2012). Such a means would promote good quality caregiving, with recent studies indicating this would reduce various costs which are placed on society (Bachmann et al., 2019). There is a significant opportunity to consider how the specific detailed assessments such as the SSAP can therefore be harnessed to inform therapeutic practice. Ensuring the centrality of relationships that looked-after children have with carers both in residential and foster care will allow for the most positive impacts for the children who are placed within these environments.

## Data Availability Statement

The data that support the findings of this study are available on request from the corresponding author (SH). The data are not publicly available due to restrictions (e.g., information that could compromise the identity and privacy of research participants).

## Ethics Statement

Ethical approval was authorized through UCL Research Ethics Committee Office for the Vice Provost (0389/025: The internal representations of children and adolescents in care, 2017). Informed consent for participation was obtained from the children’s local authority social workers, Five Rivers supervising social workers and from the children’s foster carers/residential care team. The study was also covered by the UCL data protection registration.

## Author Contributions

SH trained the clinicians to administer the SSAP, whilst the data generated from the SSAP was transcribed by KA and postgraduate students at University College London (UCL). SH coded the SSAP, conducted the qualitative analysis, and other postgraduate students at UCL. SH conducted the qualitative analysis. KA collated the data. SH wrote the manuscript with significant input from KA and RC.

## Conflict of Interest

The authors declare that the research was conducted in the absence of any commercial or financial relationships that could be construed as a potential conflict of interest.
